# Characterization of the key aroma compounds in cigar filler tobacco leaves from different production regions

**DOI:** 10.3389/fpls.2024.1476807

**Published:** 2024-12-16

**Authors:** Chenxi Jiang, Jinxiong Lv, Lingbo Ji, Hongyue An, Mingxuan Yang, Yang Huang, Lulu Liu, Zhongrong Jiang, Xiujuan Xu, Jun Hu

**Affiliations:** ^1^ Key Laboratory in Flavor and Fragrance Basic Research, Zhengzhou Tobacco Research Institute, China National Tobacco Corporation, Zhengzhou, China; ^2^ China Tobacco Sichuan Industrial Co., Ltd., Chengdu, China

**Keywords:** cigar, aroma compound, OAV, multivariate analysis, metabolic pathways, production region

## Abstract

Cigar tobacco leaves exhibited distinct regional characteristics, and aroma compounds were the key substances determining the different style features of cigars. However, the differences in aroma characteristics and the mechanisms of key aroma compound synthesis have not been fully elucidated. This study collected filler tobacco leaves (FTLs) from 5 representative domestic and international production regions. Gas chromatography-mass spectrometry (GC-MS) identified aroma compounds, an aroma wheel was established based on odor activity values (OAV), and principal component analysis (PCA) and orthogonal partial least squares discriminant analysis (OPLS-DA) revealed major differences. Synthesis pathways of key differential components were further explored using the Kyoto Encyclopedia of Genes and Genomes (KEGG). In this study, 56 aroma compounds were identified in FTLs. Imported-FTLs (IMP-FTLs) contained higher levels of ketones and esters, along with moderate nicotine content, and exhibited a more noticeable sour and woody aroma. In contrast, Domestic-FTLs (DOM-FTLs) had a greater distribution of aldehydes, phenols, and neophytadiene, presenting a more prominent bean, burnt-sweet, and floral aroma. Nine compounds, including sclareol, 5-methylfurfural, and (*E*)-5-isopropyl-8-methylnona-6,8-dien-2-one, were identified as key differential components, and their synthesis primarily involves pathways such as phenylalanine metabolism and carotenoid biosynthesis. These findings provided a novel perspective on the targeted enhancement of key aroma compounds, which was significant for improving the aroma quality of filler tobacco leaves.

## 1 Introduction

Cigars are highly esteemed globally for their complex and nuanced flavors. Unlike traditional cigarettes, which contain a blend of tobacco and other additives, cigars are purely made from cigar tobacco leaves (Liu et al., 2013). The tobacco leaves used in cigar production are categorized into wrapper, binder, and filler leaves based on their roles in the final product (Li et al., 2012). Filler tobacco leaves (FTLs) are the core part of a cigar, comprising approximately 85% of its weight (Cai et al., 2019). The quality of the FTLs directly affected the overall quality of the cigar products.

The evaluation of FTL quality primarily included aspects such as chemical composition (Li et al., 2024), sensory quality (Yan et al., 2021), physical properties (Lu et al., 2021), and microbial fermentation characteristics (Zhang et al., 2024a). Among these, the chemical composition, including volatile organic compounds, alkaloids, and amino acids, serves as the foundational basis for assessing quality (Zhang et al., 2024b; Wang et al., 2024a). The aroma of FTLs was influenced by various chemical components. Identifying the key aroma compounds that influence the style characteristics of FTLs has been a hotspot in flavor chemistry in recent years. He et al. (2023) found that the differential components in FTL from different regions were primarily ketones and heterocycles. Shi et al. (2023) determined that trimethylamine was a key substance distinguishing FTLs from Hubei, the Dominican Republic, and Indonesia, contributing to the pungent ammonia and fishy flavors. Hu et al. (2023) identified that ketones, aldehydes, and olefins were the key differential components distinguishing Changcheng No. 2 and Monte No. 4. These results demonstrated that key aroma compounds effectively differentiate varieties and origins of tobacco leaves, highlighting the style characteristics of FTLs.

The flavor characteristics of FTLs are closely related to the natural environment of the production region. A suitable natural environment provides the ecological foundation for high-quality tobacco leaf development. High-quality cigar tobacco leaves were produced in only a few countries, where special climatic and soil conditions contributed to the unique flavors of FTLs from different regions (Li et al., 2013). For example, Cuban cigars were known for their rich aroma and clean taste; American cigar tobacco leaves had a gentler taste with less strong flavor and fewer off-odors (Zheng et al., 2022a); Brazilian cigar tobacco leaves had caramel and herbal aromas (Zheng et al., 2022b). Due to the unique geographical conditions, several areas in southern China, such as Sichuan, Yunnan, Hubei, and Hainan, had gradually developed into significant regions for growing FTLs. However, compared to Imported-FTLs (IMP-FTLs), most Domestic-FTLs (DOM-FTLs) lack a rich aroma and have less distinctive flavor characteristics (Zhong et al., 2024). This disparity demonstrated the significant role of environmental factors in the synthesis of aroma compounds in FTLs, placing emphasis on understanding the pathways through which these factors exerted their effects.

The aroma compounds in FTLs were primarily derived from the transformation and degradation of aroma precursors during the growth and fermentation processes. Based on their sources, aroma compounds primarily include Maillard reaction products, phenylalanine degradation products, carotenoid degradation products, and abietane-type diterpenoid degradation products (He et al., 2023). Current research on FTLs has focused more on the effects of fermentation conditions (Ding et al., 2024) and tobacco leaf varieties (Lu et al., 2024) on the production of aroma compounds. Although studies on FTLs of regions have been reported, most research focused on the compositional differences and differential screening of aroma compounds. Few studies investigated the metabolic pathways of key differential components based on regional differences. The specific impact of geographical factors on aroma compound synthesis requires further study.

To further elucidate the aroma characteristics of FTLs from different regions and the impact of environmental factors on key components, FTL samples were collected from 5 domestic and international production regions. Differences in aroma compound composition between DOM-FTLs and IMP-FTLs were analyzed using gas chromatography-mass spectrometry (GC-MS), while aroma profiles were characterized using an aroma wheel. Multivariate statistical methods identified key differential components, the metabolic pathways of these components were investigated, and the impact of geographical factors on the formation of aroma compounds was discussed. The research results identified the flavor characteristics of FTLs from different regions and the synthesis mechanisms of key aroma compounds, providing a theoretical basis for targeted component regulation and flavor quality enhancement.

## 2 Materials and methods

### 2.1 Materials and reagents

FTL samples primarily came from leading cigar tobacco regions such as Indonesia, the Dominican Republic, and China’s Sichuan, Yunnan, and Hubei. These were supplied by the Great Wall Cigar Factory of China Tobacco Sichuan Industrial Co., Ltd., with details listed in **Table 1**.

**Table 1:** Details of samples.

No.	Regions	Cultivars	Grades and Positions	Samples Name	Samples Category
C1	SC	CX-2	Fi-C-2-Bt	SC-FTL	DOM-FTL
C2	SC	DX-4	Fi-C-2-Bt-M	SC-FTL	
C3	SC	DX-7	Fi-C-2-Bt-M	SC-FTL	
C4	YN	YX-1	Fi-C-2-Bt-L	YN-FTL	
C5	YN	YX-2	Fi-C-1-Bt-L	YN-FTL	
C6	YN	YX-6	Fi-C-3-Bt-L	YN-FTL	
C7	YN	YX-36	Fi-B-2-Bt-M	YN-FTL	
C8	HB	CX-14	Fi-C-2-Bt-M	HB-FTL	
C9	HB	CX-26	Fi-C-2-Bt-M	HB-FTL	
C10	HB	CX-81	Fi-C-2-Bt-M	HB-FTL	
C11	ID	Besuki No	FSMBO 1	ID-FTL	IMP-FTL
C12	D.R	Olor	Seco-14A	D.R-FTL	

DOM-FTL was referred to Domestic-FTL; IMP-FTL was referred to Imported-FTL.

Ethanol, dichloromethane, and C_7_–C_30_ n-alkanes (solvent: hexane) were obtained from Sigma-Aldrich (Shanghai, China). The derivatization reagent N, O-bis(trimethylsilyl)trifluoroacetamide (BSTFA) was obtained from J&K Scientific (Beijing, China). Details of the 48 standard substances and 2 internal standards were provided in **Supplementary Table S1**.

### 2.2 Sample preparation

Samples were prepared using the half-leaf method, whereby tobacco leaves were bisected and the main veins removed. Half of each leaf was mixed uniformly and dried in an oven at 40°C for 30 minutes. After drying, the leaves were sieved through a 40-mesh screen and placed in a constant temperature and humidity chamber (22°C, 60% humidity) for 24 hours to equilibrate before use.

Preliminary experiments indicated that dichloromethane effectively extracted acidic compounds and ensured complete derivatization with BSTFA. Therefore, dichloromethane was selected for acidic compound extraction. For polar non-acidic components, ethanol was used due to its safety and environmental advantages. In GC-MS analysis, detecting the carboxyl group (-COOH) is challenging due to its high polarity and low volatility (Hofstetter et al., 2019). Coupling silylation with GC-MS, using BSTFA as the silylation agent, significantly improved sensitivity and selectivity. Consequently, acidic compounds underwent silylation with BSTFA prior to analysis.

Extraction of acidic compounds: A 2.5 g sample was weighed into a beaker, and 50 mL dichloromethane with 48.4 μg/mL (*E*)-2-hexenoic acid as the internal standard was added. Ultrasonic-assisted extraction was performed at 600 W for 15 minutes. After extraction, solid-liquid separation was achieved using a filtration apparatus. The filtrate was obtained using a 0.45 μm organic phase membrane filter. For derivatization, 200 μL of BSTFA was added to 1 mL of the filtrate and heated at 100°C for 40 minutes. The solution was then cooled to room temperature before analysis. Each sample was prepared with three technical replicates.

Extraction of non-acidic compounds: A 5.0 g sample was weighed into a beaker, and 50 mL ethanol with 0.92 μg/mL phenethyl acetate as the internal standard was added. Ultrasonic-assisted extraction was performed at 600 W for 30 minutes. After extraction, solid-liquid separation was achieved using a filtration apparatus. The filtrate was obtained using a 0.45 μm organic phase membrane filter. Each sample was prepared with three technical replicates.

### 2.3 GC-MS analysis

According to the method of Yang et al. (2024a) with slight modifications, the aroma compounds were determined by GC-MS (8890-5977B, Agilent, Santa Clara, CA). To comprehensively cover the compounds in the sample and enhance improve analysis accuracy, both a non-polar DB-5 MS (60 m × 0.25 mm × 0.25 μm) column and a polar DB-WAXETR column (60 m × 0.25 mm × 0.25 μm) were employed.

Acidic compounds: The DB-5MS capillary column was used for analysis. High-purity helium (99.999%) served as the carrier gas at a flow rate of 1 mL/min. The temperature program started at 50°C and ramped at 4°C/min to a final temperature of 280°C, where it was held for 5 minutes. Ionization was performed with an EI ion source at 70 eV and a source temperature of 230°C. The mass spectrometer operated in full scan mode with a range of 33–455 amu, while selected compounds were quantified using ion-monitoring mode.

Non-acidic compounds: Both the DB-5MS and DB-WAXETR capillary columns were used. For the DB-5MS column, the temperature program started at 50°C and ramped at 3°C/min to a final temperature of 280°C, where it was held for 10 minutes. For the DB-WAXETR capillary columns, the temperature program started at 50°C and ramped at 3°C/min to a final temperature of 230°C, where it was held for 10 minutes. All other conditions were identical to those used for acidic compounds.

### 2.4 Identification of aroma compound

The retention index (RI) was determined using n-alkanes (C_7_–C_30_). Compounds were identified by comparing their mass spectral data to the NIST 17 database (match threshold>85%) and their RI values to those reported in the literature, including the NIST Chemistry Webbook (https://webbook.nist.gov/chemistry/). For compounds without calculable RI values, identification was based on retention time matching through standard injections.

### 2.5 Standard calibration curve

Stock solutions of mixed standards (excluding acidic compounds) were prepared in absolute ethanol, while the stock solution of acidic compounds was prepared in dichloromethane, and then diluted to 7 different concentrations. The same amount of internal standard used in the FTL samples was added to the standard solutions, which were then analyzed by GC-MS under the same conditions as the sample analysis.

Calibration curves were constructed by plotting the ratio of the target aroma compound peak area to the internal standard peak area against the concentration ratio. The quantification ions, calibration curve equations, linearity ranges limits of detection (LOD) and recovery were presented in **Table 2**.

**Table 2:** Quantitative ion, standard curve, linearity ranges limits of detection (LOD) and recovery of aroma compounds.

No.	CAS	Compounds	Quantitative ion (m/z)	Standard curve	R^2^	LOD (μg/L)	Recovery (%)
1	100-52-7	Benzaldehyde	106	y=0.7950x+5.5986*10^-4^	0.999 4	6.88	101
2	432-25-7	*β*-Cyclocitral	152	y=0.3083x+5.8768*10^-4^	0.999 9	12.28	109
3	620-02-0	5-Methylfurfural	110	y=0.4196x-0.0076	0.999 3	1.14	107
4	928-68-7	6-Methyl-2-heptanone	58	y=0.4641x-3.2138*10^-4^	0.999 9	2.04	114
5	110-93-0	6-Methyl-5-hepten-2-one	108	y=0.2870x-0.0056	0.999 3	8.09	97
6	497-23-4	2(5*H*)-Furanone	55	y=0.0249x+8.9745*10^-5^	0.999 5	2.37	101
7	122-00-9	4’-Methylacetophenone	119	y=0.5093x+0.0844	0.999 1	3.92	97
8	14901-07-6	*β*-Ionone	177	y=0.6076x-0.0019	0.999 5	4.08	105
9	141-79-7	Mesityl oxide	83	y=0.6009x+1.6648*10^-4^	0.999 2	5.54	100
10	98-86-2	Acetophenone	105	y=1.2808x-2.8022*10^-4^	0.999 8	7.09	98
11	1604-28-0	6-Methyl-3,5-heptadien-2-one	109	y=0.6928x+1.4905*10^-4^	0.999 7	3.96	108
12	78-59-1	Isophorone	82	y=0.9103x-0.0038	0.999 1	3.58	101
13	502-69-2	6,10,14-Trimethyl-2-pentadecanone	58	y=0.2447x+0.0118	0.999 8	181.68	107
14	35044-68-	*β*-Damascone	177	y=0.0162x-1.8453*10^-5^	0.999 9	9.66	102
15	3796-70-1	Geranylacetone	43	y=0.1575x+0.0035	0.999 3	23.99	109
16	473-08-5	*α*-Cyperone	218	y=0.1708x-5.3291*10^-5^	0.999 6	7.59	94
17	1125-21-9	2,6,6-Trimethyl-2-cyclohexene-1,4-dione	68	y=0.3946x-0.0029	0.999 2	4.13	102
18	762-29-8	Farnesylacetone	69	y=0.0462x-0.0097	0.999 1	21.14	104
19	503-74-2	Isoamyl acid	60	y=0.7998x+0.0128	0.999 1	41.13	97
20	105-43-1	3-Methylvaleric acid	75	y=0.7980x+0.0044	0.999 6	3.50	114
21	50-21-5	Lactic acid	73	y=0.6892x+0.0073	0.999 2	2.78	103
22	65-85-0	Benzoic acid	179	y=4.4118x+8.6316*10^-4^	0.999 1	12.86	113
23	124-07-2	Octanoic acid	201	y=1.1403x+8.8017*10^-4^	0.999 9	4.95	94
24	110-15-6	Succinic acid	147	y=2.3294x-9.6223*10^-4^	0.999 1	3.41	93
25	121-34-6	Vanillic acid	73	y=0.7510x-0.0062	0.999 2	8.51	104
26	544-63-8	Myristic acid	285	y=0.5432x-0.0045	0.999 2	6.00	94
27	101-97-3	Ethyl phenylacetate	91	y=1.1114x+0.0014	0.999 7	14.24	95
28	17092-92-1	Dihydroactinidiolide	111	y=0.5865x-0.0084	0.999 2	12.83	107
29	96-48-0	*γ*-Butyrolactone	42	y=0.0684x-0.0056	0.999 3	19.63	105
30	564-20-5	Sclareolide	123	y=0.0924x+0.0527	0.999 2	198.08	95
31	98-00-0	Furfuryl alcohol	98	y=0.2568x-8.2965*10^-4^	0.999 4	4.63	96
32	60-12-8	Phenethyl alcohol	91	y=0.6924x+0.0357	0.999 2	22.30	104
33	102608-53-7	3,7,11,15-Tetramethyl-2-hexadecen-1-ol	81	y=4.5484x+2.3353*10^-4^	0.999 0	23.75	104
34	505-32-8	Isophytol	71	y=0.3475x-0.0582	1.000 0	156.84	103
35	515-03-7	Sclareol	177	y=0.3721x-0.0395	0.999 4	14.45	97
36	110-86-1	Pyridine	79	y=0.5516x-0.0087	0.999 8	29.53	96
37	109-06-8	2-Picoline	93	y=0.6307x-0.0036	0.999 5	11.81	107
38	108-48-5	2,6-Lutidine	107	y=1.9846x-2.4109*10^-4^	0.999 2	1.92	99
39	108-99-6	3-Picoline	93	y=0.8271x+0.0081	0.999 2	6.05	99
40	636-41-9	2-Methyl-1*H*-pyrrole	80	y=0.7485x+0.0044	0.999 1	3.19	94
41	350-03-8	3-Acetylpyridine	106	y=0.3188x-4.2069*10^-4^	0.999 7	26.02	106
42	1008-88-4	3-Phenylpyridine	155	y=1.0015x-0.0761	0.999 9	100.21	91
43	120-72-9	Indole	117	y=1.0164x+0.0146	0.999 4	163.28	98
44	5989-27-5	(+)-Limonene	68	y=0.4521x+0.0039	0.999 2	6.73	102
45	504-96-1	Neophytadiene	68	y=0.0879x+0.0375	0.999 1	1908.81	112
46	7786-61-0	2-Methoxy-4-vinylphenol	150	y=0.3730x+0.0153	0.999 3	24.05	101
47	90-05-1	Guaiacol	109	y=0.3780x+0.0011	0.999 1	5.92	99
48	100679-85-4	(-)-Ambroxide	221	y=1.1318x-0.0082	0.999 6	10.30	102

### 2.6 Quantitation of aroma compounds

The concentrations of aroma compounds were calculated using calibration curves. For compounds without suitable standards, semi-quantitative analysis was performed based on the peak area and the known concentration of the internal standard (i.e., phenethyl acetate or (*E*)-2-hexenoic acid). The concentration of each compound was calculated using the following equation:


$${\text{ρ}}_{a}=\frac{A_{a}}{{\text{A}}_{\text{IS}}}\times \frac{{\text{C}}_{\text{IS}}{\text{V}}_{\text{IS}}}{\text{M}}$$

where ${\text{ρ}}_{\text{a}}$ was the content of the target compound in μg/g; A_a_ was the peak area of the target compound; A_IS_ was the peak area of the internal standard; C_IS_ was the known concentration of the internal standard in μg/μL; V_IS_ was the volume of the internal standard added in μL; M was the weight of the cigar tobacco leaves in g.

### 2.7 Odor activity values

The olfactory thresholds were obtained from the literature (Sun, 2010; Van Gemert, 2018; Yang et al., 2020; Shi et al., 2023). The OAV of each compound was calculated using the following equation:


$$\text{OA}{\text{V}}_{\text{i}}=\frac{\text{Ci}}{\text{Ti}}$$

where C_i_ was the content of compound (μg/g) and T_i_ was the detection threshold of compound in water (mg/kg).

### 2.8 Statistical analysis

Sample differences were analyzed using SPSS (v26.0, Chicago, IL, USA) via one-way analysis of variance (ANOVA) and Duncan’s approach, with P<0.05 considered significant. The relationships among aroma compounds were evaluated by calculating Spearman’s rank order correlation coefficients. The heatmap was generated using TBtools-II (v2.096) (Chen et al., 2023). Principal Component Analysis (PCA) and orthogonal partial least-squares discriminant analysis (OPLS-DA) were performed in Simca (v14.1), with differential compounds identified by variable importance in projection (VIP) >1 and P<0.05. Pathway enrichment analysis of differential aroma compounds was conducted in MetaboAnalyst 5.0 (https://www.metaboanalyst.ca/) using P<0.05 as the threshold for significant enrichment. The metabolic pathways of differential aroma compounds were identified via the Kyoto Encyclopedia of Genes and Genomes (KEGG) (https://www.kegg.jp/kegg/pathway.html).

## 3 Results

### 3.1 Composition of aroma compounds in FTLs

**Figure 1:**
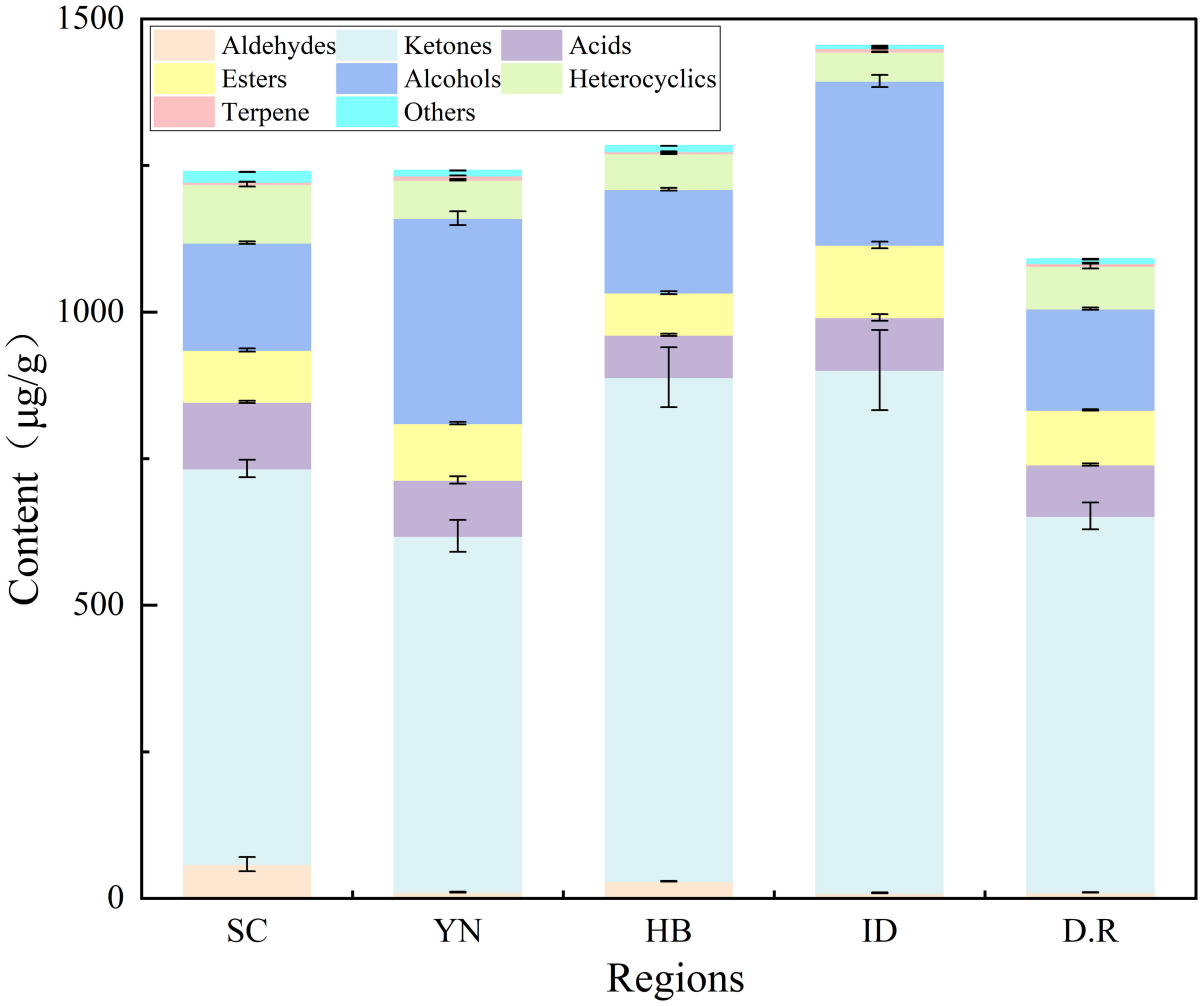
Aroma compounds content in FTLs from different regions.

The qualitative results of the samples were shown in **Supplementary Table S2**. A total of 56 aroma compounds were identified and screened for all the FTL samples, comprising 19 ketones, 6 alcohols, 4 esters, 9 acids, 3 aldehydes, 9 heterocycles, 3 terpenes, and 3 other types. As shown in **Figure 1**, **Table 3**, the total content of aroma compounds in FTLs ranged from 1090.14 to 1454.23 μg/g across various production regions (excluding nicotine and neophytadiene), with minimal variation observed among the domestic regions. The highest content was found in ID-FTLs, while the lowest was in D.R-FTLs.

**Table 3:** Contents of aroma compounds in the FTLs from different production regions.

NO.	Compounds	Content (μg/g)				
		SC-FTL	YN-FTL	HB-FTL	ID-FTL	D.R-FTL
1	Benzaldehyde	1.03 ± 0.04c	1.61 ± 0.02a	1.17 ± 0.03b	1.02 ± 0.12c	1.02 ± 0.10c
2	5-Methylfurfural	57.04 ± 12.21a	8.66 ± 0.69c	27.8 ± 0.64b	8.18 ± 0.58c	8.87 ± 0.41c
3	*β*-Cyclocitral	0.28 ± 0.02a	0.29 ± 0.01a	0.28 ± 0.01a	0.27 ± 0.02a	0.22 ± 0.01b
Aldehydes (3)		58.34 ± 12.26a	10.57 ± 0.71c	29.25 ± 0.63b	9.47 ± 0.71c	10.11 ± 0.41c
4	6-Methyl-2-heptanone	0.9 ± 0.06a	0.58 ± 0.01b	0.66 ± 0.00b	0.61 ± 0.06b	0.46 ± 0.09c
5	6-Methyl-5-hepten-2-one	11.64 ± 0.13b	10.08 ± 0.11d	12.86 ± 0.13a	11.02 ± 0.22c	11.69 ± 0.36b
6	2(5*H*)-Furanone	16.15 ± 0.58a	4.49 ± 1.01c	10.59 ± 0.39b	1.41 ± 0.14e	2.59 ± 0.44d
7	4’-Methylacetophenone	4.52 ± 0.20a	2.4 ± 0.11c	3.46 ± 0.08b	3.52 ± 0.33b	1.68 ± 0.14d
8	*β*-Ionone	2.55 ± 0.11b	3.48 ± 0.05a	2.8 ± 0.04b	3.33 ± 0.34a	1.96 ± 0.07c
9	Mesityl oxide	3.1 ± 0.14d	6.93 ± 0.49ab	6.05 ± 0.10b	7.5 ± 0.41a	4.47 ± 0.91c
10	Acetophenone	0.47 ± 0.07b	1.26 ± 0.06a	0.15 ± 0.00c	0.09 ± 0.00c	0.1 ± 0.00c
11	6-Methyl-3,5-heptadien-2-one	0.06 ± 0.00a	0.04 ± 0.01b	0.06 ± 0.00a	0.02 ± 0.00c	0.02 ± 0.00c
12	Isophorone	2.1 ± 0.04b	1.77 ± 0.01d	2.19 ± 0.05a	1.72 ± 0.04d	1.88 ± 0.01c
13	2,6,6-Trimethyl-2-cyclohexene-1,4-dione	4.38 ± 0.22c	4.45 ± 0.06c	5.33 ± 0.22a	4.97 ± 0.24b	3.65 ± 0.05d
14	(*E*)-5-Isopropyl-8-methylnona-6,8-dien-2-one	76.15 ± 2.47a	62.25 ± 4.94b	50.76 ± 2.98c	78.16 ± 7.46a	46.74 ± 1.31c
15	*β*-Damascone	30.91 ± 2.26a	19.05 ± 1.10c	20.72 ± 1.76bc	14.73 ± 1.34d	22.26 ± 0.24b
16	Geranylacetone	30.1 ± 2.00b	17.24 ± 0.91c	34.03 ± 2.46ab	35.39 ± 1.15a	32.29 ± 3.55ab
17	4,7,9-Megastigmatrien-3-one II	15.83 ± 0.72a	10.99 ± 0.81b	15.86 ± 1.17a	8.61 ± 0.93c	2.83 ± 0.07d
18	3-Hydroxy-*β*-damascone	10.77 ± 0.55a	4.77 ± 0.30c	8.64 ± 0.70b	3.86 ± 0.24d	7.81 ± 0.20b
19	4,7,9-Megastigmatrien-3-one IV	13.93 ± 0.68a	9.81 ± 0.48b	14.7 ± 1.12a	6.56 ± 0.24c	2.04 ± 0.04d
20	*α*-Cyperone	1.32 ± 0.10a	0.61 ± 0.00c	0.98 ± 0.04b	0.53 ± 0.03c	0.35 ± 0.02d
21	6,10,14-Trimethyl-2-pentadecanone	69.44 ± 3.62c	97 ± 4.70ab	103.81 ± 8.33a	97.53 ± 8.79ab	87.67 ± 5.68b
22	Farnesylacetone	380.56 ± 5.04bc	350.41 ± 14.34c	565.87 ± 32.81a	611.95 ± 48.29a	411.68 ± 12.64b
Ketones (19)		674.87 ± 14.92b	607.59 ± 27.23b	859.52 ± 51.08a	891.52 ± 68.33a	642.15 ± 22.78b
23	Isoamyl acid	5.37 ± 0.21c	11.59 ± 0.40a	5.71 ± 0.19c	9.32 ± 0.49b	9.48 ± 0.10b
24	3-Methylvaleric acid	8.17 ± 1.02c	18.19 ± 1.05b	8.09 ± 0.23c	35.75 ± 2.10a	16.46 ± 0.04b
25	Lactic acid	14.84 ± 1.36a	2.63 ± 0.22b	2.53 ± 0.12b	2.64 ± 0.06b	2.39 ± 0.12b
26	Benzoic acid	7.08 ± 0.24b	9.04 ± 0.71a	5.38 ± 0.20d	2.64 ± 0.15e	6.13 ± 0.26c
27	Octanoic acid	0.39 ± 0.02a	0.17 ± 0.02b	0.4 ± 0.01a	0.19 ± 0.04b	0.22 ± 0.03b
28	Phenylacetic acid	39.96 ± 0.07a	27.27 ± 2.64b	17.91 ± 0.54c	10.48 ± 0.79d	29.06 ± 1.24b
29	Succinic acid	5.05 ± 0.46a	3.11 ± 0.20b	2.03 ± 0.09c	0.48 ± 0.02e	1.04 ± 0.12d
30	Vanillic acid	8.26 ± 0.73a	4.53 ± 0.14c	6.03 ± 0.16b	3.88 ± 0.07c	5.59 ± 0.16b
31	Myristic acid	24.36 ± 0.97a	18.94 ± 1.19b	24.11 ± 0.60a	24.33 ± 2.06a	17.25 ± 1.57b
Acids (9)		113.46 ± 1.92a	95.46 ± 6.33b	72.2 ± 1.94c	89.7 ± 5.53b	87.61 ± 2.03b
32	Ethyl phenylacetate	0.29 ± 0.04a	0.2 ± 0.02b	0.19 ± 0.01cd	0.14 ± 0.02d	0.2 ± 0.03b
33	Dihydroactinidiolide	24.75 ± 2.29c	20.52 ± 0.21d	17.87 ± 0.57d	42.02 ± 1.38a	32.02 ± 2.29b
34	*γ*-Butyrolactone	45.03 ± 0.20a	34.37 ± 0.36c	38.93 ± 0.59b	31.25 ± 0.22d	30.44 ± 0.01e
35	Sclareolide	18.45 ± 0.35d	41.71 ± 1.97b	15.06 ± 1.52d	50.39 ± 4.55a	30.5 ± 1.18c
Esters (4)		88.52 ± 2.66c	96.81 ± 2.25b	72.05 ± 2.67d	123.79 ± 5.78a	93.16 ± 1.1bc
36	Furfuryl alcohol	25.27 ± 2.71a	8.12 ± 0.12c	17.6 ± 0.67b	4.63 ± 0.12d	15.81 ± 1.36b
37	Phenethyl alcohol	15.15 ± 1.34a	8.02 ± 0.19b	8.03 ± 0.21b	4.06 ± 0.07d	5.49 ± 0.52c
38	3,7,11,15-Tetramethyl-2-hexadecen-1-ol	0.12 ± 0.02b	0.09 ± 0.00bc	0.06 ± 0.00c	0.21 ± 0.04a	0.1 ± 0.01b
39	Isophytol	70.65 ± 1.23c	72.76 ± 1.02b	72.2 ± 0.61bc	75.99 ± 1.33a	72.88 ± 0.50b
40	Thunbergol	22.31 ± 0.86c	202.1 ± 10.14a	23.97 ± 2.66c	132.25 ± 8.17b	13.9 ± 0.79c
41	Sclareol	49.63 ± 0.18d	58.62 ± 2.28b	54.59 ± 0.78c	62.46 ± 1.18a	64.5 ± 0.58a
Alcohols (6)		183.13 ± 2.07c	349.71 ± 11.73a	176.45 ± 2.42c	279.61 ± 10.38b	172.68 ± 2.03c
42	Pyridine	22.98 ± 0.71a	12 ± 0.98c	14.22 ± 0.98b	8.47 ± 0.07d	11.64 ± 0.41c
43	2-Picoline	2.41 ± 0.03a	2.3 ± 0.01bc	2.34 ± 0.01b	2.21 ± 0.00d	2.28 ± 0.04c
44	2,6-Lutidine	0.16 ± 0.00a	0.08 ± 0.00d	0.1 ± 0.00b	0.09 ± 0.00bc	0.09 ± 0.00cd
45	3-Picoline	0.83 ± 0.06a	0.4 ± 0.01	0.43 ± 0.00bc	0.28 ± 0.02d	0.48 ± 0.05b
46	2-Methyl-1*H*-pyrrole	1.64 ± 0.13a	0.67 ± 0.10c	1.36 ± 0.01b	0.34 ± 0.04d	0.55 ± 0.01c
47	3-Acetylpyridine	6.51 ± 0.26bc	7.84 ± 0.40b	6.95 ± 0.41bc	6.31 ± 0.69c	11.27 ± 1.23a
48	3-Phenylpyridine	30.52 ± 0.04b	29.52 ± 0.07d	29.17 ± 0.07d	30.13 ± 0.19c	33.15 ± 0.37a
49	Indole	34.64 ± 4.56a	12.67 ± 0.59b	6.64 ± 0.23c	1.99 ± 0.35d	13.25 ± 2.07b
50	Nicotine	12962.65 ± 610.11b	20856.78 ± 896.87a	22507.65 ± 1241.94a	7878.03 ± 375.63c	14897.88 ± 1385.19b
Heterocycles (9)		13062.34 ± 612.46b	20922.26 ± 896.21a	22568.86 ± 1243.02a	7927.85 ± 375.19c	14970.59 ± 1388.83b
51	(+)-Limonene	2.52 ± 0.31b	2.31 ± 0.20b	2.61 ± 0.13b	2.77 ± 0.51b	4.05 ± 0.48a
52	Cembrene	1.51 ± 0.03c	4.94 ± 0.26a	0.8 ± 0.04d	3.26 ± 0.47b	0.96 ± 0.07d
53	Neophytadiene	7232.73 ± 351.53ab	7359.62 ± 240.67a	7430.88 ± 249.25a	6752.77 ± 270.3ab	6511.65 ± 572.86b
Terpenes (3)		7236.75 ± 351.75ab	7366.87 ± 240.67a	7434.29 ± 249.22a	6758.8 ± 271.23ab	6516.67 ± 572.73b
54	2-Methoxy-4-vinylphenol	11.53 ± 0.47a	3.77 ± 0.24c	5.25 ± 0.07b	0.6 ± 0.04e	2.11 ± 0.24d
55	Guaiacol	2.43 ± 0.41a	1.56 ± 0.31b	1.21 ± 0.04b	0.65 ± 0.05c	1.48 ± 0.27b
56	(-)-Ambroxide	2.99 ± 0.06bc	3.01 ± 0.03bc	2.93 ± 0.03c	3.03 ± 0.03ab	3.11 ± 0.05a
Others (3)		16.95 ± 0.33a	8.34 ± 0.12c	9.39 ± 0.05b	4.29 ± 0.09e	6.7 ± 0.56d
Total (56) (Excluding nicotine and neophytadiene)		1238.98 ± 4.13b	1241.22 ± 47.33b	1283.47 ± 58.65b	1454.23 ± 80.98a	1090.14 ± 24.92c

Letters within the same row did not differ significantly at the 95% confidence level.

Ketones, alcohols, acids, esters, and aldehydes were the main aroma compounds in these FTLs, consistent with the results reported by Zhu et al. (2024). The highest content of ketones was found in ID-FTLs, primarily consisting of (*E*)-5-isopropyl-8-methylnona-6,8-dien-2-one, geranylacetone, and farnesylacetone contributions. These compounds were primarily degradation products of plastid pigments, significantly affecting the aroma and sensory quality of tobacco (Gao et al., 2014). YN-FTLs and ID-FTLs were characterized by higher alcohol content, primarily due to the high content of thunbergol. As one of the cembrane degradation products, thunbergol possessed a light orange fragrance (Wang et al., 2023a). Aldehydes were found to be more concentrated in SC-FTLs and HB-FTLs, predominantly attributable to the contribution of 5-methylfurfural, which imparts a caramel aroma (Ge et al., 2020). Among all samples, ID-FTLs contained a higher concentration of esters. The primary constituents identified were dihydroactinidiolide and sclareolide, which exhibited musky and woody aromas, respectively. Heterocycles were primarily derived from the Maillard reaction and exhibit roasted, baked, and nutty aromas (Shi et al., 2008). The heterocycle content in SC-FTLs was significantly higher than in other samples. Notable compounds, including pyridine and indole, were often linked to unpleasant odors. The content of acidic compounds in SC-FTLs was notably high. It is noteworthy that the lactic acid content in SC-FTLs was approximately seven times that of other samples. While its direct impact on aroma was minimal, it can make the smoke smoother and more refined (Yang et al., 2024b). Although concentrations of phenols and ethers were minor in FTLs, they were found to exert a considerable influence on their flavor profile. The content of 2-methoxy-4-vinylphenol in SC-FTLs was significantly higher than in other samples, primarily exhibiting a smoky aroma. High concentrations of indole and 3-methylindole, often associated with unpleasant odors, were typically masked by this strong smoky aroma (Chi et al., 2019).

As an important intermediate product of chemical reactions in tobacco, neophytadiene not only directly affected the sensory quality of tobacco leaves but also influenced the formation and degradation of other aroma components (Wang et al., 2021). Neophytadiene had the highest content among the terpene compounds, consistent with previous research findings (Shi et al., 2006). The content in DOM-FTLs was higher than in IMP-FTLs. Nicotine was noted for its physiological actions, which include stimulating the central nervous system and alleviating fatigue (Afolalu et al., 2021). A moderate amount of nicotine provides smokers with appropriate physiological stimulation, pleasant aroma, and smooth taste (Zhang et al., 2020). In contrast, an excessive amount of nicotine could produce a pungent odor that adversely affects the flavor of tobacco leaves (Wang et al., 2023b). The lowest nicotine content was observed in ID-FTLs, while YN-FTLs exhibited the highest levels.

### 3.2 Characterization of FTL aroma profile from different regions using OAV

The OAV integrates both the content and perception threshold of aroma compounds, providing a quantitative measure of their contribution to the FTL aroma profile. Compounds with an OAV<1 are usually considered potential aroma components, while those with an OAV>1 are generally regarded as having a certain contribution to the overall aroma. When the OAV exceeds 10, the substance is considered to significantly contribute to the overall flavor characteristics. Based on the theory, the calculation results for the OAV of aroma compounds were shown in **Table 4**. A total of 36 compounds with OAV>1 were detected, which can be considered aroma-active compounds in the FTLs. In all samples, *β*-damascone, 4,7,9-megastigmatrien-3-one II, 4,7,9-megastigmatrien-3-one IV, (-)-ambroxide, *β*-ionone, 2-methoxy-4-vinylphenol, and guaiacol exhibited OAV significantly higher than other aroma compounds. These results aligned with the findings of Wang et al. (2024b), suggesting that these compounds were crucial to the FTL aroma. The SC-FTLs exhibited the highest OAV, indicating their stronger aromatic effect compared to other regions. Moreover, the D.R-FTLs featured the highest number of compounds with OAV>1, which implied a greater complexity in aroma characteristics. This was consistent with the results reported by Zheng et al. (2022a).

**Table 4:** OAV of aroma-active compounds in the FTLs.

NO.	Compounds	Threshold(mg/kg)	Description	OAV				
				SC-FTL	YN-FTL	HB-FTL	ID-FTL	D.R-FTL
1	Benzaldehyde	0.75089	Nutty, cherry	1	2	2	1	1
2	5-Methylfurfural	1.11	Burnt-sweet, bitter almond	51	8	25	7	8
3	*β*-Cyclocitral	0.003	Herbal, woody	94	98	94	90	73
4	6-Methyl-2-heptanone	0.0081	Fruity	111	72	82	75	56
5	6-Methyl-5-hepten-2-one	0.068	Citrus	171	148	189	162	172
6	4’-Methylacetophenone	0.021	Bean, fruity and floral	215	114	165	167	80
7	*β*-Ionone	0.0035	Floral, violet	729	994	801	953	559
8	Acetophenone	0.065	Coumarin	7	19	2	1	2
9	2,6,6-Trimethyl-2-cyclohexene-1,4-dione	0.025	Fresh, sweet	175	178	213	199	146
10	(E)-5-Isopropyl-8-methylnona-6,8-dien-2-one	1.82	Carrot, licorice and tea-like	42	34	28	43	26
11	*β*-Damascone	0.001	Floral, sweet	309 07	190 49	207 21	147 34	222 55
12	Geranylacetone	0.06	Floral, tobacco	502	287	567	590	538
13	4,7,9-Megastigmatrien-3-one II	0.00205	Tobacco, spicy	772 1	536 3	773 6	420 1	138 1
14	4,7,9-Megastigmatrien-3-one IV	0.00088	Tobacco, spicy	158 27	111 43	167 09	745 8	232 3
15	Isoamyl acid	0.49	Sour	11	24	12	19	19
16	3-Methylvaleric acid	0.046	Sour, sweet	178	395	176	777	358
17	Benzoic acid	1	Styrax benzoin	7	9	5	3	6
18	Phenylacetic acid	6.1	Burnt sugar, honey	7	4	3	2	5
19	Myristic acid	10	Balsamic, creamy	2	2	2	2	2
20	Ethyl phenylacetate	0.15555	Floral, sweet and fruity	2	1	1	<1	1
21	Dihydroactinidiolide	3.8	Fruity, musk	7	5	5	11	8
22	*γ*-Butyrolactone	1	Creamy, bean	45	34	39	31	30
23	Furfuryl alcohol	4.5005	Burnt-sweet	6	2	4	1	4
24	Phenethyl alcohol	0.56423	Floral, sweet	27	14	14	7	10
25	Sclareol	8	Amber	6	7	7	8	8
26	Pyridine	2	Ammonia, fishy	11	6	7	4	6
27	2-Picoline	0.05	Toasty	48	46	47	44	46
28	2,6-Lutidine	0.003	Bread-like, raw green and nutty	54	27	33	30	29
29	3-Picoline	0.042	Hazelnut, earthy	20	10	10	7	11
30	2-Methyl-1*H*-pyrrole	0.0006	Fatty	274 1	112 5	225 9	563	924
31	3-Acetylpyridine	0.5	Nutty	13	16	14	13	23
32	Indole	0.011	Floral, bad odor at high content	314 9	115 1	604	181	120 4
33	(+)-Limonene	0.034	Citrus, mint	74	68	77	81	119
34	2-Methoxy-4-vinylphenol	0.01202	Sweet, smoky and clove	959	314	437	50	176
35	Guaiacol	0.00084	Smoky, spicy	289 7	186 1	144 3	778	175 8
36	(-)-Ambroxide	0.0003	Woody	998 1	100 20	975 2	101 16	103 59
	Total			767 97	526 52	622 82	414 11	427 25

**Figure 2:**
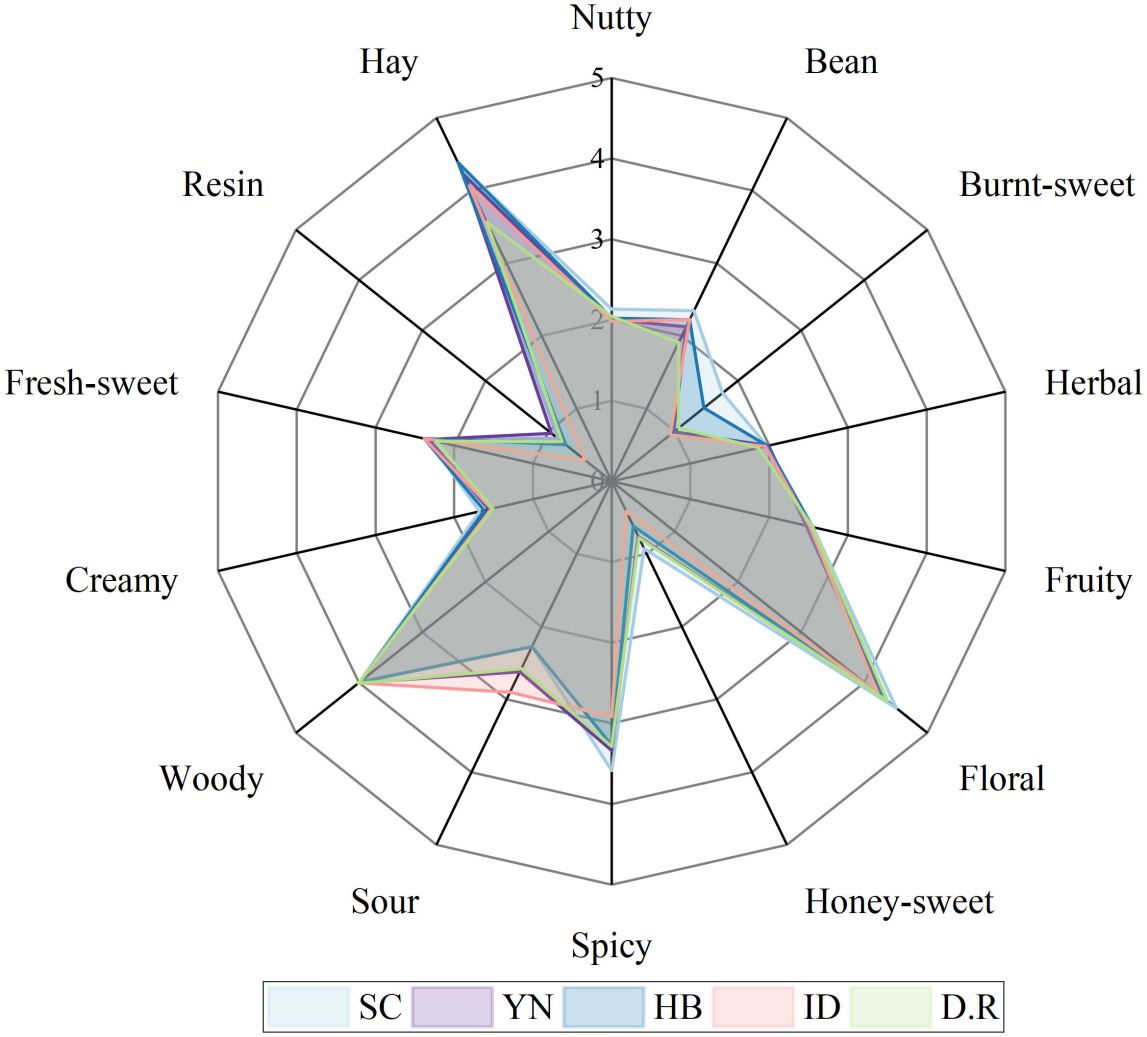
Aroma wheel of FTLs from different regions.

To more accurately depict the aroma profiles of FTLs, the analysis was conducted by constructing aroma wheels. All compounds were divided into 14 categories based on their odor descriptions, which include nutty, bean, burnt-sweet, herbal, fruity, floral, honey-sweet, spicy, sour, woody, creamy, fresh-sweet, resin, and hay. The OAV for each odor property was derived from the total OAVs of compounds within that category. Compounds with an OAV<1 can still contribute to aromas through the cumulative effects of structurally or olfactorily similar compounds (Francis Newton, 2005). Consequently, their contributions were not disregarded in this analysis. The sum of OAV for each odor property was calculated logarithmically, and the results were presented on an aroma wheel. All the aroma wheel radar charts of the FTL samples were shown in **Figure 2**. Higher levels of hay, floral, and woody scents were exhibited by all FTLs aroma wheels, along with typical spicy and sour notes. SC-FTLs had the strongest nutty, bean, burnt-sweet, and honey-sweet aromas, consistent with the findings of Zhong et al. (2024). The hay aroma was prominent in HB-FTLs, while D.R-FTLs exhibited a stronger woody aroma. Overall, DOM-FTLs exhibited stronger bean, burnt-sweet, floral, and spicy aromas, while sour and woody aromas were more prominent in IMP-FTLs.

Among them, the representative compounds of floral included *β*-damascone, geranylacetone, phenethyl alcohol, and *β*-ionone, which were the basic aroma compounds of FTL with OAV>1. With the highest OAV, *β*-damascone was the major contributor to the floral scent in all samples. The representative compound for the hay aroma was primarily 4,7,9-megastigmatrien-3-one. As an important aroma compound in tobacco, 4,7,9-megastigmatrien-3-one significantly enhanced the aroma, harmonized the taste, reduced irritability, and mainly contributed to the inherent tobacco flavor and spicy (Xie, 2009). Nutty was one of the characteristic flavors of FTLs, primarily contributed by 2-picoline, 2,6-lutidine, 3-acetylpyridine, and 3-picoline. It was noteworthy that not all heterocyclic compounds produced pleasant aromas. For instance, 2-methyl-1*H*-pyrrole had a greasy scent; indole emitted a jasmine fragrance at low concentrations but an unpleasant odor at higher ones; and pyridine intensified the sharp ammonia smell due to its fishy odor. Because the three compounds frequently exhibited unpleasant odors, they were excluded from the categorizations of the odor properties. 2-Methoxy-4-vinylphenol and guaiacol played a crucial role due to their low odor detection thresholds and were representative compounds of the spicy aroma. Burnt-sweet was primarily contributed by 5-methylfurfural and furfuryl alcohol, which were significant products of the Maillard reaction. The representative compounds of the sour aroma were isoamyl acid and 3-methylvaleric acid, both of which were important volatile organic acids in tobacco. 4’-Methylacetophenone and (-)-ambroxide were present at low contents in FTL but contributed significantly to the bean and woody aromas. Although the OAV of other aromas was not prominent, they also enhanced the richness of the FTL aroma.

### 3.3 Distribution characteristics of aroma-active compounds in FTL from different production regions

**Figure 3:**
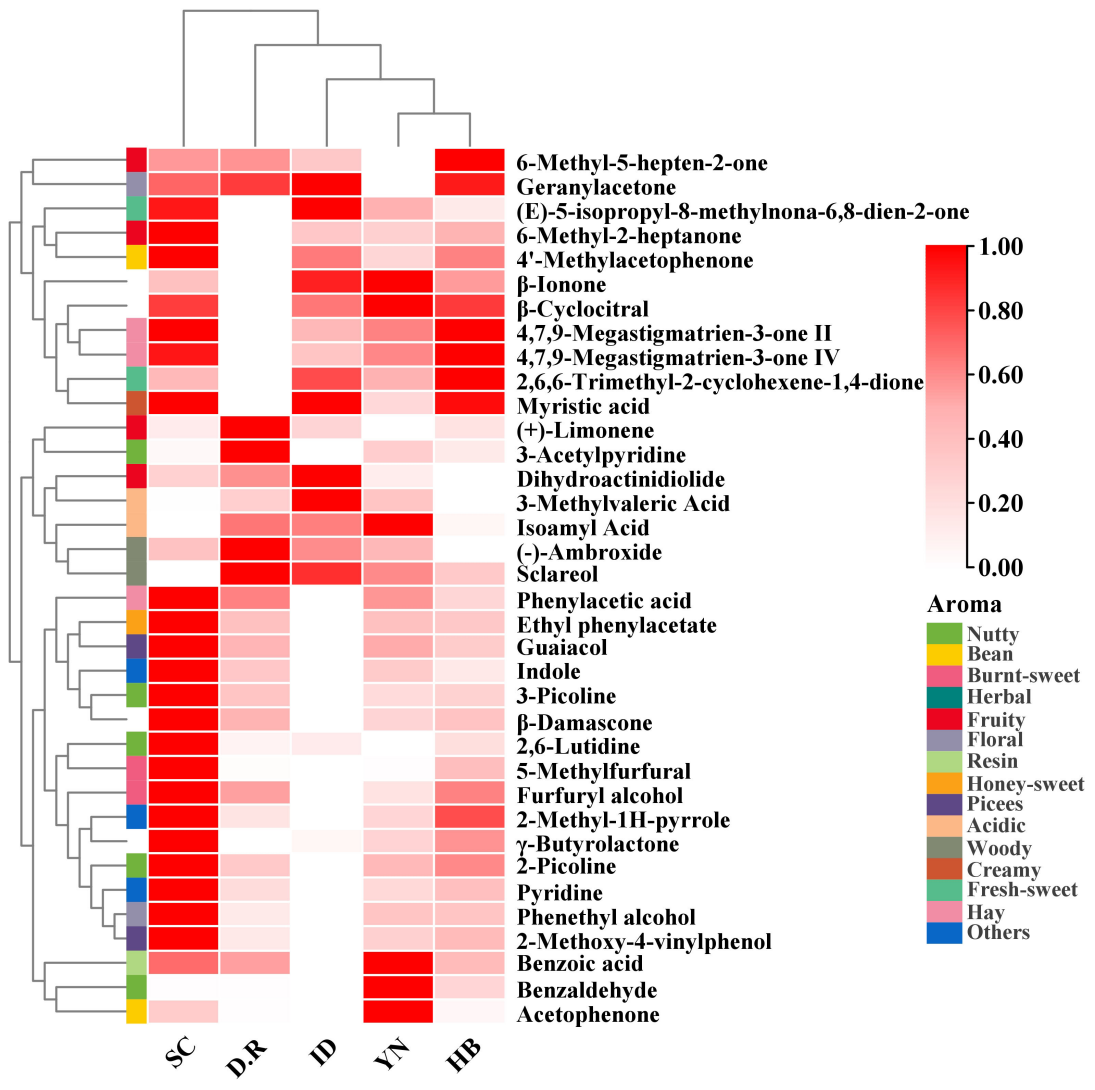
Clustering heatmap of aroma-active compounds in FTLs from different regions.

The clustering heatmap based on 36 aroma-active compounds in FTLs from different production regions was shown in **Figure 3**. The clustering heatmap divided 36 aroma-active compounds into 5 categories, highlighting regional variations in FTL’s aroma compound distribution. Phenylacetic acid, ethyl phenylacetate, guaiacol, indole, 3-picoline, *β*-damascone, 2,6-lutidine, 5-methylfurfural, and other 16 compounds were present at higher concentrations in SC-FTLs than in other FTLs, boosting its rich and intense aroma. The YN-FTLs and HB-FTLs exhibited only minor differences compared to IMP-FTLs. Analyses revealed that the concentrations of *β*-cyclocitral, *β*-ionone, 4,7,9-megastigmatrien-3-one II, 4,7,9-megastigmatrien-3-one IV, and 2,6,6-trimethyl-2-cyclohexene-1,4-dione were comparable between HB-FTLs and IMP-FTLs. In contrast, YN-FTLs and IMP-FTLs had higher levels of isoamyl acid, 3-methylvaleric acid, (-)-ambroxide, and sclareol, significantly enhancing their sour and woody aromas. It is noteworthy that YN-FTLs contained more benzoic acid, benzaldehyde, and acetophenone—three phenylalanine degradation products that respectively imparted resin, nutty, and bean aromas to the FTLs—compared to other groups.

### 3.4 Multivariate statistical analysis of aroma compounds in FTLs

Pearson correlation analysis was conducted on 56 aroma compounds, as shown in **Supplementary Table S3**. Highly significant positive correlations were observed among various compounds. Notably, (1) pyridine, furfuryl alcohol, 2(5*H*)-furanone, 2-methoxy-4-vinylphenol, *γ*-butyrolactone, isophorone, and 3-hydroxy-*β*-damascone were found to be significantly correlated (P<0.01); (2) vanillic acid, pyridine, furfuryl alcohol, 2-methoxy-4-vinylphenol, *γ*-butyrolactone, isophorone, and 3-hydroxy-*β*-damascone also exhibited significant correlations; (3) 2-picoline, pyridine, 2-methyl-1*H*-pyrrole, 2(5*H*)-furanone, and 2-methoxy-4-vinylphenol showed similar significant correlations. Negative correlations among compounds were also observed. In addition, 21 substances exhibited minimal correlation with other aroma compounds. Owing to their potential impact on subsequent multivariate statistical analyses, these substances were excluded from further analyses as variables.

**Figure 4:**
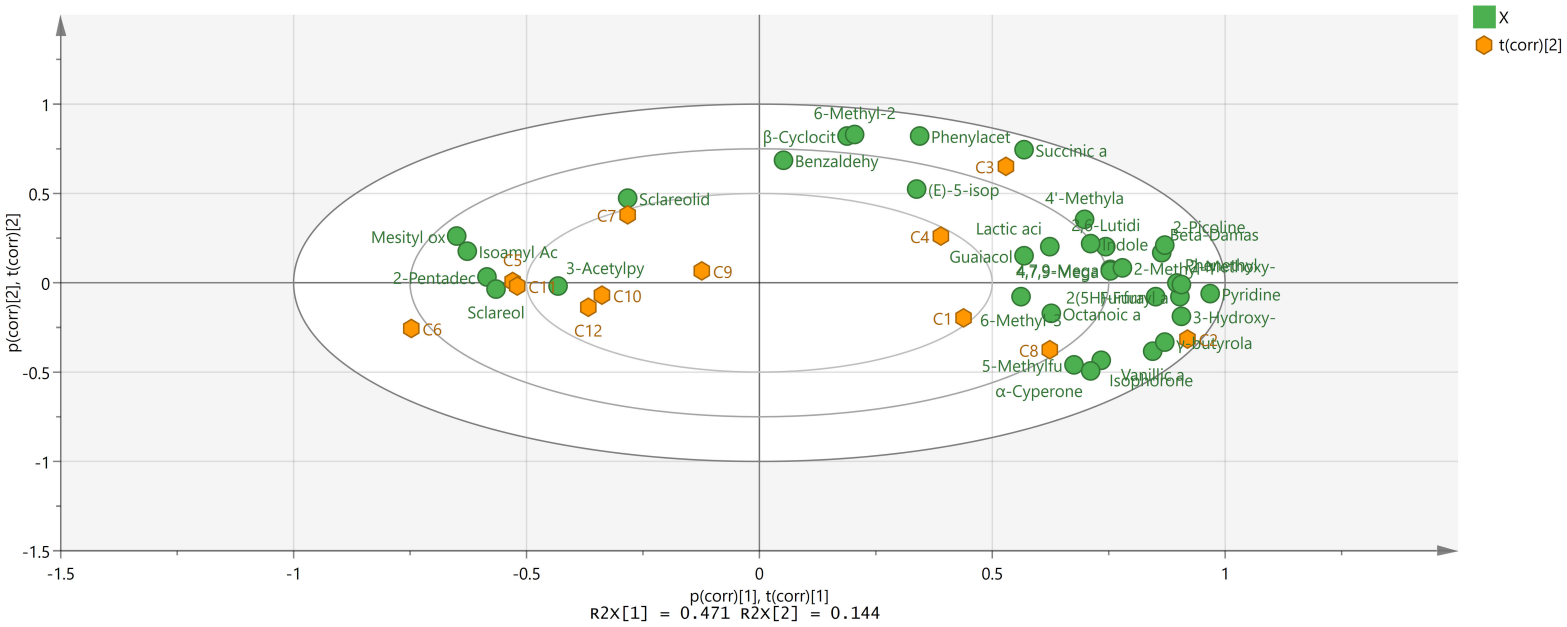
PCA Biplot of FTLs from different regions.

In order to reduce the number of variables for analysis, PCA employs dimensionality reduction. To achieve precise and visually clear classification outcomes, 35 aroma compounds with significant correlations were selected for PCA. The outcomes were shown in **Figure 4**. Four principal components were chosen for the investigation using the PCA model. PC1 contributed 47.1% of the variation, which was significantly more than the other components. PC2 and PC3 each contributed 14.4% and 11.2% of the variance, respectively. The three major components accounted for 72.7% of the overall variance observed in FTLs. This suggested that the constructed PCA model had a high predictive power. It was noted that 12 samples were divided into two groups based on the PCA score plot. According to the distribution across various regions, the Sichuan region formed a distinct cluster, but the Yunnan and Hubei regions clustered with the two international regions. These findings showed that HB-FTLs and YN-FTLs were more similar to IMP-FTLs in terms of the distribution features of aroma-active compounds. The PCA biplot further displayed data from the loading plot, where the proximity of points on the graph denoted the association between FTL samples and aroma compounds. SC-FTLs were mainly related to 6-methyl-3,5-heptadien-2-one, vanillic acid, *γ*-butyrolactone, 3-hydroxy-*β*-damascone, and succinic acid, indicating that these substances contributed more to SC-FTLs. YN-FTLs were more closely linked to guaiacol, (*E*)-5-isopropyl-8-methylnona-6,8-dien-2-one, sclareol, and sclareolide, which may be its distinctive fragrance components. HB-FTLs showed close relationships with isophorone, 5-methylfurfural, 3-acetylpyridine, and *α*-cyperone. IMP-FTLs were more closely associated with (*E*)-5-isopropyl-8-methylnona-6,8-dien-2-one, sclareol, and 3-acetylpyridine. Notably, YN-FTLs and IMP-FTLs shared the greatest number of distinctive compounds, which could account for their similarities.

**Figure 5:**
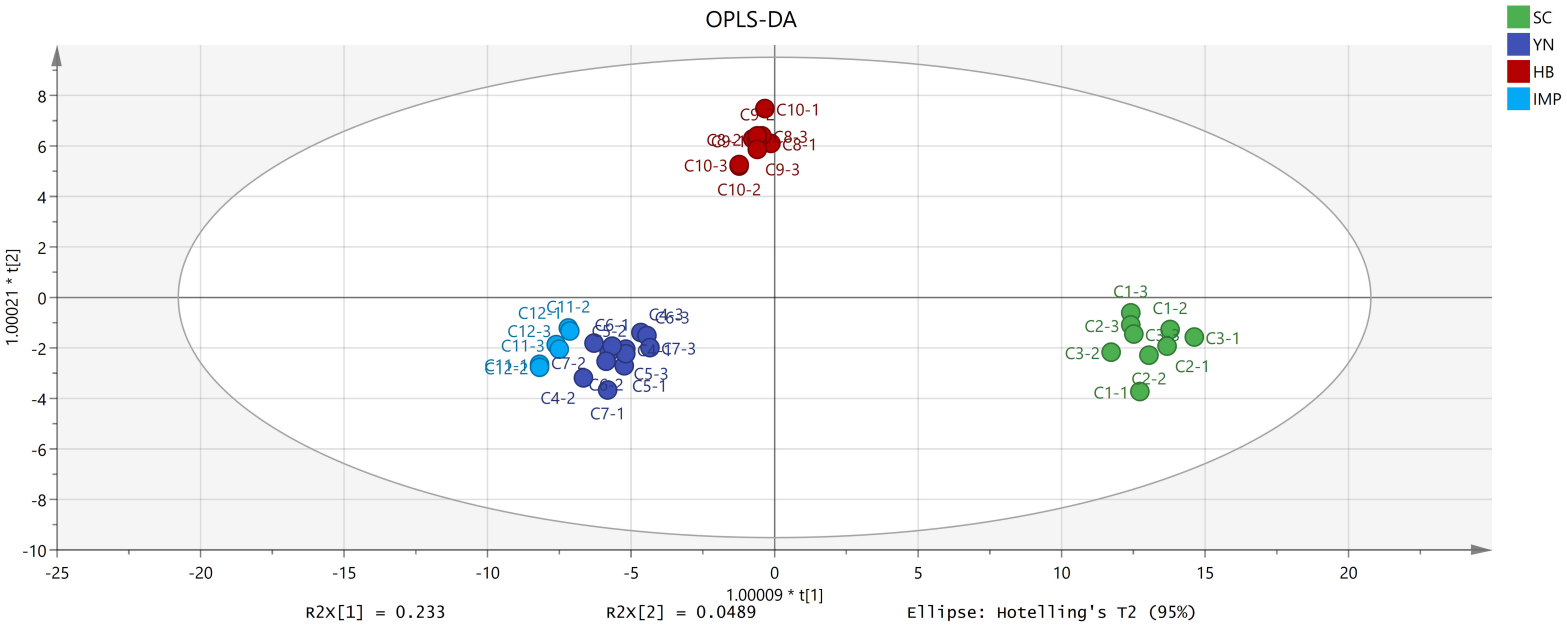
OPLS-DA score plot of FTLs from different regions.

An OPLS-DA analysis was established to identify differential compounds in FTLs from various production regions. The analysis focused on 35 relevant aroma compounds, selecting components based on their VIP values. Based on the OPLS-DA model generated (**Figure 5**), it was observed that the fit index for the independent variable (R^2^X) reached 0.999, the fit index for the dependent variable (R^2^Y) achieved 0.989, and the predictive index (Q^2^) was determined to be 0.874. R^2^ and Q^2^ exceed 0.5, indicating that the model fit results were acceptable. The FTLs from different regions were classed separately into four clusters, and the overall classification appearance was well. Large differences within the class resulted from their diverse contents of differential aroma components. Notably, YN-FTLs and IMP-FTLs were closely aligned but remained distinguishable, indicating smaller differences between them. After that, there was a growing divergence between them, with HB-FTLs being closer to IMP-FTLs and SC-FTLs being the furthest away.

**Figure 6:**
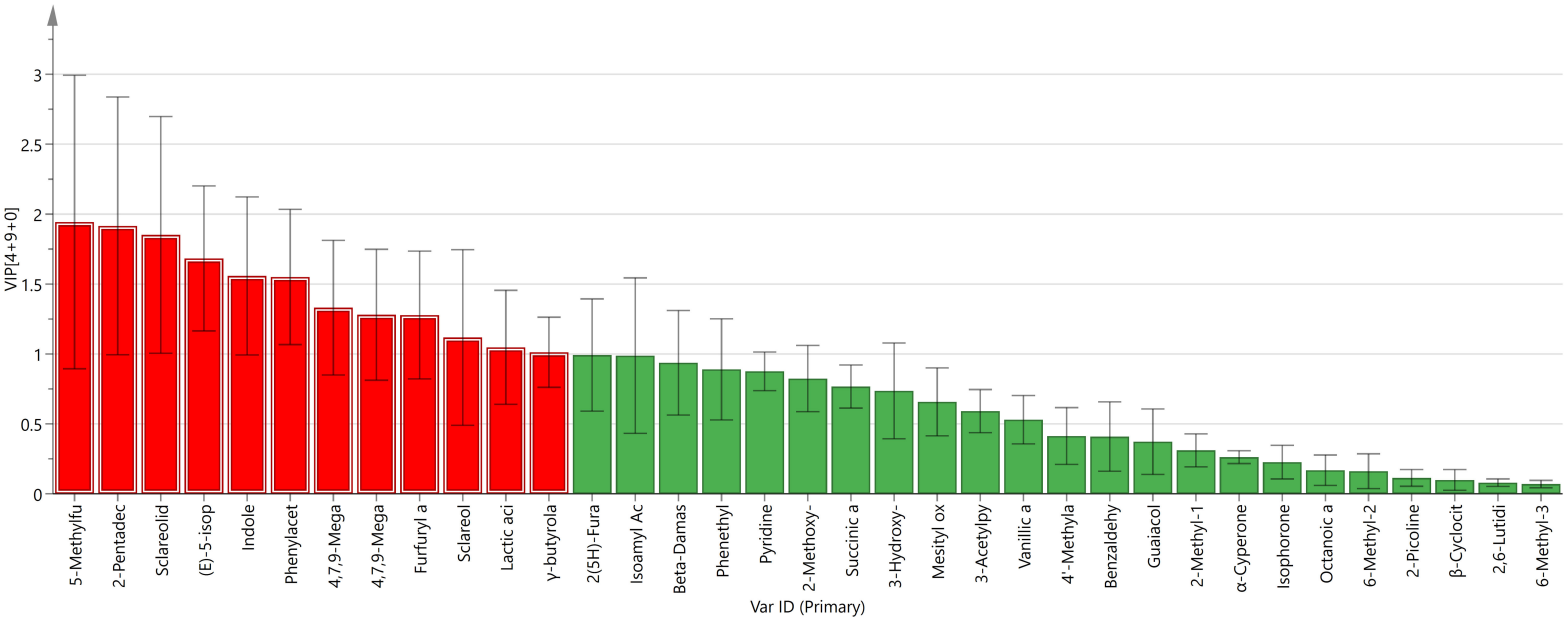
Compounds satisfying VIP >1 of FTLs from different regions.

**Figure 7:**
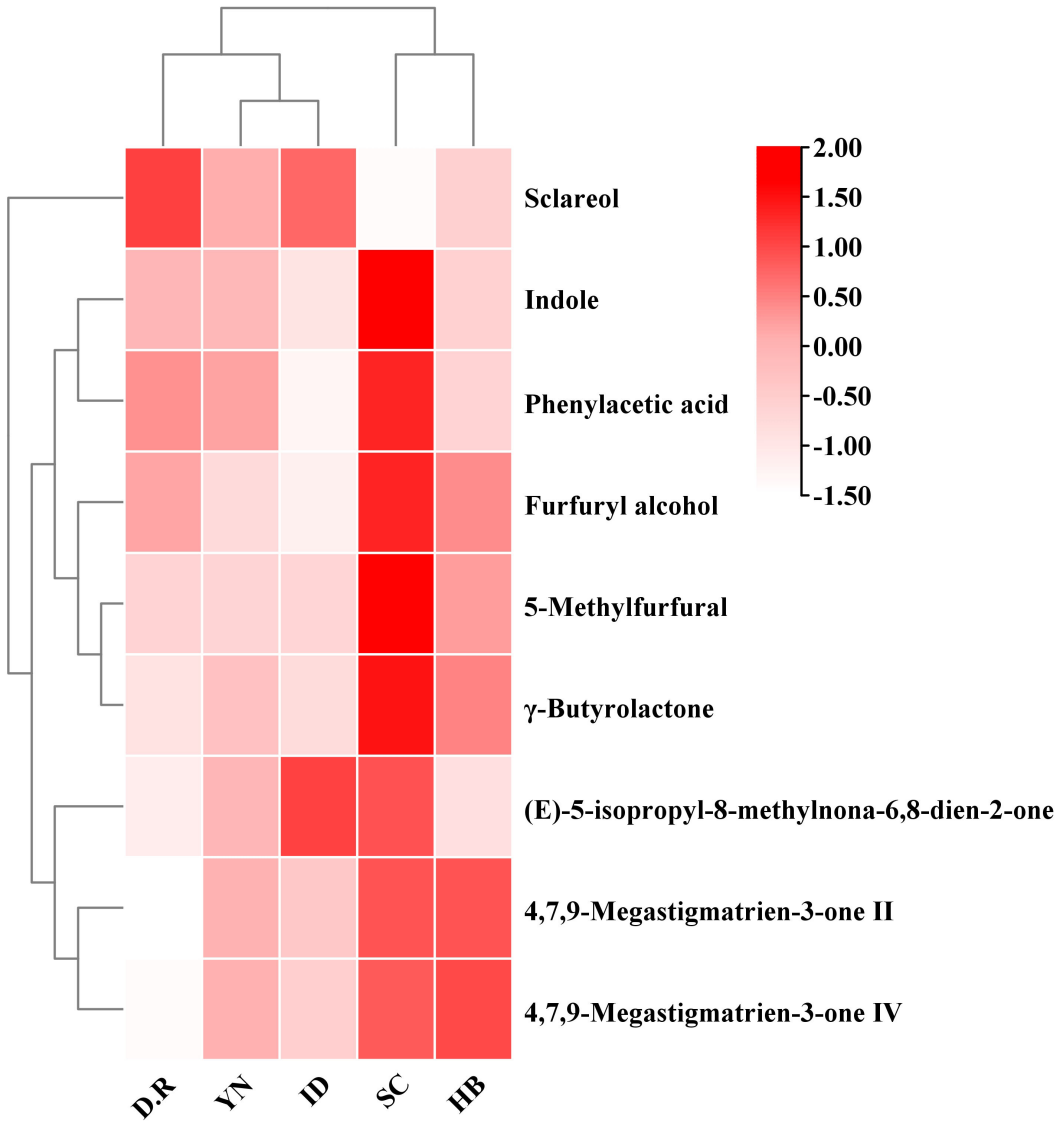
Clustering heatmap of key differential compounds in FTLs from different regions.

As shown in **Figure 6**, 12 differential compounds were screened based on P < 0.05 and VIP >1. Combining OAV>1, 9 key differential compounds were obtained, and their clustering heatmap was shown in **Figure 7**. The five regions were separated into two groups based on the clustering in the heatmap: YN and the two imported regions in one group, and SC and HB in the other. The clustering results were in accord with the OPLS-DA findings. In addition to sclareol, SC-FTLs had a wider distribution of other key differential components. Overall, the most prominent components in DOM-FTLs were 4,7,9-megastigmatrien-3-one II, 4,7,9-megastigmatrien-3-one IV, *γ*-butyrolactone, 5-methylfurfural, and furfuryl alcohol, while the most prominent components in IMP-FTLs were sclareol. Most of these substances had pleasant aromas and played an important role in harmonizing, modifying, and differentiating the overall aroma of FTLs from different regions.

### 3.5 Analysis of the metabolic pathways of key differential compounds

The degradation of precursors such as sugars, pigments, amino acids, polyphenols, and alkaloids was the primary source of aroma compounds in tobacco (Liu et al., 2022). Through the analysis of key differential compounds in FTL from different regions, it was found that most of these compounds belonged to phenylalanine metabolism, the biosynthesis of phenylalanine, tyrosine, and tryptophan, and carotenoid biosynthesis, as shown in **Table 5**. Two pathways (P<0.05) were identified as primary factors influencing the biosynthesis of key differential compounds. Although the metabolic pathways of sclareol were not enriched in MetaboAnalyst, they were obtained from KEGG and were also presented in the diagram.

**Table 5:** Enrichment results of metabolic pathways.

NO.	Pathway Name	Match Status	P value	Impact value	Database
1	Phenylalanine metabolism	1/12	0.023 8	0.000	KEGG
2	Phenylalanine, tyrosine and tryptophan biosynthesis	1/22	0.043 4	0.000	KEGG
3	Carotenoid biosynthesis	1/43	0.083 6	3.5E^-4^	KEGG

**Figure 8:**
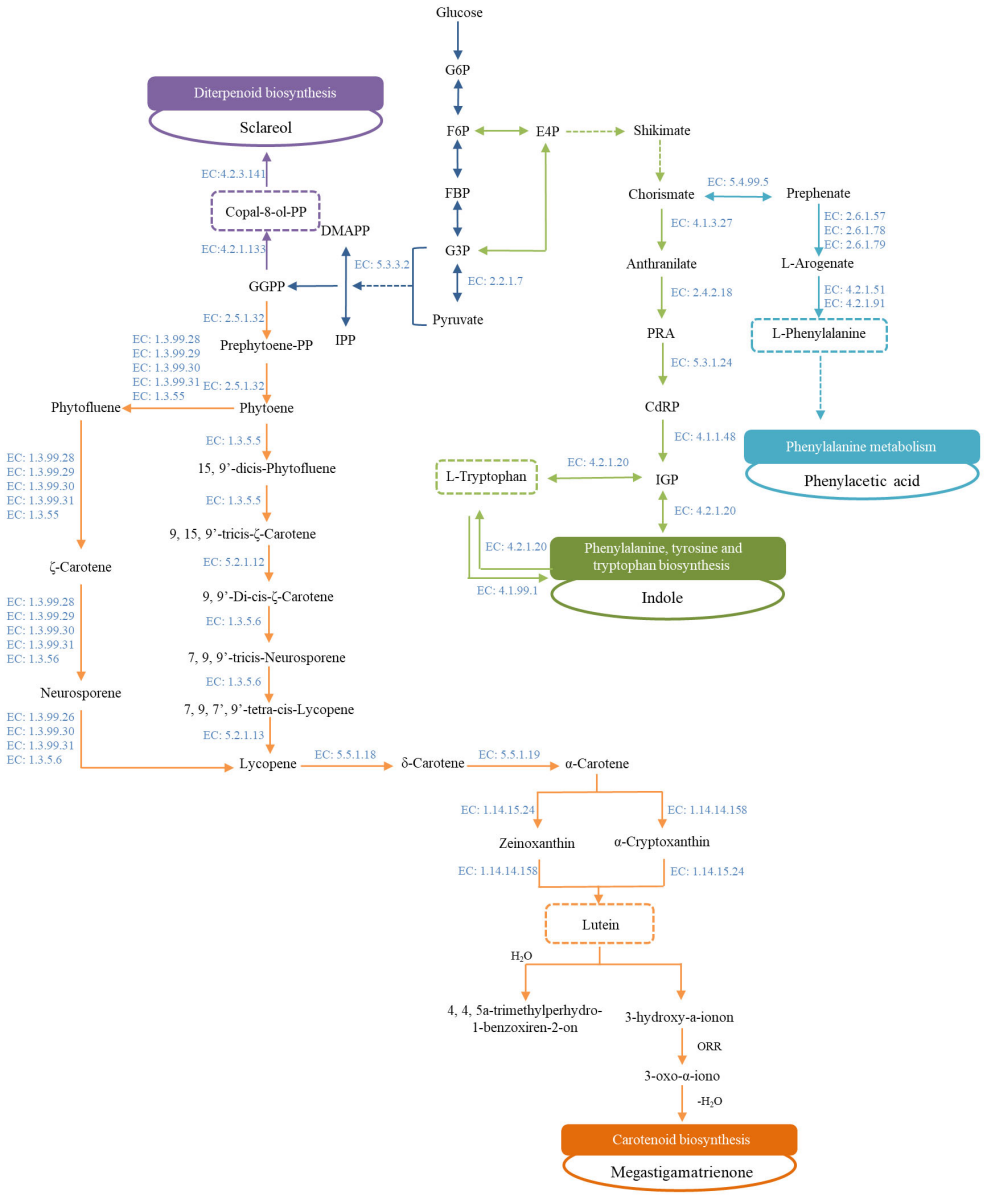
Metabolic network of the key differential compounds in FTLs. G6P, *α*-D-Glucose 6-phosphate; F6P, D-Fructose 6-phosphate; FBP, D-Fructose 1,6-bisphosphate; G3P, D-Glyceraldehyde 3-phosphate; DMAPP, Dimethylallyl diphosphate; IPP, Isopentenyl diphosphate; GGPP, Geranylgeranyl diphosphate; E4P, D-Erythrose 4-phosphate; PRA, N-(5-Phospho-D-ribosyl) anthranilate; CdRP, 1-(2-Carboxyphenylamino)-1-deoxy-D-ribulose 5-phosphate; IGP, Indoleglycerol phosphate.

As shown in **Figure 8**, the metabolic pathways and the key enzymes involved in them were illustrated. The condensation of isopentenyl diphosphate (IPP) and dimethylallyl diphosphate (DMAPP) into geranylgeranyl diphosphate (GGPP) was catalyzed by the essential enzyme geranylgeranyl pyrophosphate synthase (EC 2.5.1.1). A portion of GGPP was catalyzed by copal-8-ol diphosphate hydratase (EC: 4.2.1.133) to generate Copal-8-ol diphosphate (Copal-8-ol, PP), an important precursor of sclareol. The degradation of Copal-8-ol, PP was primarily catalyzed by sclareol synthase (EC: 4.2.3.141). The content and composition of aroma precursors were influenced not only by the biosynthetic pathway but also by degradation pathways (Zhang et al., 2024a). Additionally, GGPP was also used for the synthesis of carotenoids. 15-*cis*-Phytoene synthase (EC: 2.5.1.32) catalyzed the condensation of two GGPP molecules in the first step of the carotenoid pathway, which produced phytoene, the main carotenoid. Following a sequence of enzymatic reactions, lycopene cyclization produced *β*-carotene and *α*-carotene, which were further synthesized into lutein, a precursor to 4,7,9-megastigmatrien-3-one. Therefore, geranylgeranyl pyrophosphate synthase played a crucial role in the generation of aroma compounds, and regulating the flow of GGPP was an essential approach for enhancing aroma.

Amino acid degradation pathways were essential in the formation of cigar tobacco leaves flavor. The aromatic amino acids were formed through the shikimate pathway, which was followed by the branched aromatic amino acids biosynthesis pathway, with chorismate serving as a significant intermediate branch point metabolite (Tzin and Galili, 2010). Indole, an important product of tryptophan metabolism, not only possessed a distinctive aroma but also functioned as a scaffold for various receptors in plant cells (Sun et al., 2023). Indole-based chemicals like indole-3-butyric acid (IBA) and indoleacetic acid (IAA) were frequently employed as plant growth regulators (Chen et al., 2020; Li et al., 2018). As an important precursor of aroma compounds in tobacco leaves, phenylalanine underwent self-reaction to decompose into various aroma substances, such as benzaldehyde and benzyl alcohol (Ying et al., 2022). The process by which phenylalanine degraded to produce phenylacetic acid was more complex, and two main pathways had been identified to influence phenylacetic acid synthesis (Cook, 2019). Phenylalanine ammonia-lyase (PAL) was the rate-limiting enzyme in the phenylalanine metabolic pathway, catalyzing the first step of the pathway, and was widely present in plants (Li et al., 2016). The synthesis of L-phenylalanine was reverse-catalyzed by phenylalanine aminolyase under specific conditions (Zhao, 2016). Consequently, regulating the levels and expressions of PAL was a key strategy to control the formation of aroma compounds derived from phenylalanine degradation.

## 4 Discussion

As an important economic crop, tobacco has been known for its broad adaptability and strong plasticity. Variations in environmental conditions across growing regions influenced the growth processes of tobacco plants, thereby affecting the quality of the tobacco leaves. This study found that the compounds contributing to the aroma characteristics of FTL primarily involved key metabolic pathways, including amino acid degradation, carotenoid degradation, and diterpene metabolism. The significant differences in the flavor characteristics of FTLs from representative domestic and international production regions may be related to the regulation of these metabolic pathways by regional environmental factors. This study further examines the key metabolic pathways and their interactions with environmental conditions.

In this study, the representative compounds for bean aroma (4’-methylacetophenone and acetophenone), floral aroma (phenethyl alcohol), honey-sweet aroma (phenylacetic acid), and spicy aroma (2-methoxy-4-vinylphenol and guaiacol) were all identified as phenylalanine degradation products and their downstream metabolites. Li et al. (2017) found that the phenylalanine content and the activity of key enzymes in tobacco decreased as the environmental temperature increased. Indonesia and the Dominican Republic, both with tropical climates, had significantly higher average temperatures during the growing period compared to the three domestic regions. These findings may partially explain why DOM-FTLs contained higher levels of phenylalanine degradation products.

Carotenoids degraded under enzymatic or light conditions to produce various aroma compounds, such as *β*-damascone and *β*-ionone, which significantly influenced the flavor quality and color of tobacco (Meléndez-Martínez et al., 2023). In the carotenoid biosynthesis pathway, lycopene *β*-cyclase (EC 5.5.1.19) was the key enzyme that promoted the conversion of lycopene to *β*-carotene and *α*-carotene, which could further produce various ketone aroma compounds (Wang et al., 2024a). Previous studies have shown that *β*-cyclase abundance in Indonesian CTLs was higher, resulting in more active metabolic pathways involved in the production of *α*-carotene and *β*-carotene (Wang et al., 2024a). This finding differs from the results of this study. Liu et al. (2013) determined that light exposure facilitated the degradation of carotenoids but inhibited their accumulation. Higher-quality leaves were only produced in environments with more frequent cloud cover and less intense sunlight (Li et al., 2013). In the Indonesian growing regions, an average of 30% to 40% of daytime hours during the tobacco leaf growing period were overcast or rainy, making shade nets unnecessary for field production due to the suitable light conditions (Qin et al., 2012). The synthesis of carotenoids in Indonesian tobacco leaves was promoted by the activity of *β*-cyclase, but the light conditions during growth inhibited the degradation of carotenoids. This may explain the relatively lower content of carotenoid degradation products in Indonesian FTLs.

The representative compounds for woody aroma, sclareol and (-)-ambroxide, were identified as diterpenes and their derivatives. Terpenoids were mostly secondary metabolites of plants and possess pleasant woody and herbal aromas. Abiotic stresses associated with climate had an important impact on the concentration of terpenoids in plants. Under drought-stress conditions, isoprene emissions did not decrease with increasing drought stress (Holopainen et al., 2013). The plants released isoprene in a burst when watering reactivated the production of isoprene, which was dependent on photosynthesis (Loreto and Schnitzler, 2010). DOM-FTLs grew from May to September, during the rainy season, whereas IMP-FTLs grew from January to June (Lin et al., 2023), were planted in the dry season, and were harvested during the wet season. Differences in the rainy seasons during the growing period may have caused variations in the terpenoid concentration of FTLs. It is noteworthy that the important precursors for the synthesis of diterpenes and carotenoids were derived from the MEP pathway, where GGPP was the key enzyme in this stage (Zhang et al., 2021b). The rational design of GGPP effectively enhanced the photosynthetic efficiency of tobacco and promoted the production of carotenoids (Dong et al., 2022). Therefore, it could be considered to design and engineer key enzymes in important metabolic pathways to achieve targeted regulation of desired aroma compounds.

The climate of the production region and the style characteristics of tobacco leaves were closely related (Geng et al., 2023). The OPLS-DA and clustering heatmap results of key differential components indicated that Yunnan clustered with the two imported regions, suggesting that Yunnan shared similar ecological foundations with Indonesian and Dominican. Lin et al. (2023) analyzed the growing period climates of 13 major domestic cigar tobacco regions were more similar to those of Jember, Indonesia, and Santiago, Dominican. In this study, the samples were selected from more concentrated domestic regions with stronger regional characteristics, which may account for the differences from previous results. Given that climate conditions were likely a major driver of these ecological similarities, it was important to incorporate meteorological factors to further investigate the climate characteristics and similarities among production regions.

In addition to the environment of origin, the fermentation process was also an important factor influencing the formation of flavor compounds in tobacco leaves. Inorganic elements, enzymes, and microorganisms in the tobacco leaves work together to promote the traditional fermentation process (Zhang et al., 2024a). As shown in **Figure 8**, microorganisms influenced the synthesis of key differential components by regulating the activity of relevant enzymes. Different FTLs possessed distinct functional microorganisms, which resulted in different aroma characteristics in the fermented FTLs (Yan et al., 2022). This was an uncontrollable natural process that was significantly impacted by the environment. Consequently, exogenous substances were commonly used in industry to assist in the secondary fermentation of tobacco leaves. The fermentation process was directed toward the desired result by this approach, which theoretically influenced the growth and metabolism of microbial communities and altered biochemical reaction pathways (Zhang et al., 2021a). Research has shown that adding “Humi” during the fermentation process effectively promoted non-enzymatic Browning reactions, increased aroma compounds, reduced undesirable components, and enhanced microbial activity (Ren et al., 2024). Hu et al. (2022) added an exogenous additive primarily composed of rice wine, fritillaria cirrhosa, and loquat wine, which increased the total nitrogen, nicotine, total sugar, and aroma compound content in CTLs. The FTL samples used in this study were not subjected to industrial fermentation processes. Therefore, it could be considered to select or design appropriate exogenous additives to perform secondary fermentation, enhancing the flavor characteristics of FTLs.

## 5 Conclusion

In summary, our results systematically characterized the aroma compounds composition and aroma profile of FTLs, identified key differential components, and explored the metabolic pathways and geographical factors influencing these compounds. This study found that FTLs from different production regions, both domestic and international, exhibited significant variations in the composition of aroma compounds. The aroma wheel results showed that DOM-FTLs had more bean, burnt-sweet, flower, and spicy scents, while IMP-FTLs had more sour and woody aromas. Moreover, 9 components were identified as key differential compounds distinguishing FTLs from different regions. The ecological factors of the producing regions have an influence on the metabolic pathways of the key differential components. This study provided data support for investigating the flavor quality differences of FTLs and laid a foundation for optimizing domestic cultivation conditions and targeted regulating key aroma compounds. To further analyze the impact of regional factors on aroma compound formation, meteorological factors could be used to compare climate similarities between production regions. Additionally, exploring local geographic advantages and clarifying the ecological characteristics of domestic regions would offer a theoretical basis for developing the distinctive style of DOM-FTLs.

## Data Availability

The original contributions presented in the study are included in the article/**Supplementary Material**. Further inquiries can be directed to the corresponding authors.
