# Supplementary material for: Characterization of the key aroma compounds in cigar filler tobacco leaves from different production regions
Source: Front Plant Sci. 2024 Dec 16;15:1476807. doi: 10.3389/fpls.2024.1476807 (PMC11683104; doi:10.3389/fpls.2024.1476807)
Supplement: Supplementary file 1 [file Table1.docx]

| Standard substances | Supplier |
| --- | --- |
| 2-Picoline, 2,6-lutidine, 3-picoline, 5-methylfurfural, furfuryl alcohol, 6-methyl-5-hepten-2-one, 2(5*H*)-furanone, 4'-methylacetophenone, ethyl phenylacetate, 3-acetylpyridine, *β*-ionone, acetophenone, *β*-damascone, (-)-ambroxide, sclareol, myristic acid, isoamyl acid, 3-methylvaleric acid, isophorone, sclareolide, 3,7,11,15-tetramethyl-2-hexadecen-1-ol, 2,6,6-trimethyl-2-cyclohexene-1,4-dione, isophytol, *α*-cyperone, 3-phenylpyridine, and (*E*)-2-hexenoic acid | Sigma-Aldrich (Shanghai, China) |
| 2-Methoxy-4-vinylphenol, dihydroactinidiolide, indole, *γ*-butyrolactone, geranylacetone, pyridine, (+)-limonene, mesityl oxide, 6-methyl-3,5-heptadien-2-one, octanoic acid, vanillic acid, phenethyl alcohol, benzoic acid, 2-methyl-1*H*-pyrrole, and phenethyl acetate | Macklin (Shanghai, China) |
| 6,10,14-Trimethyl-2-pentadecanone,  neophytadiene, 6-methyl-2-heptanone, and *β*-cyclocitral | J&K Scientific (Beijing, China) |
| Farnesylacetone (mixture of isomers), lactic acid, succinic acid, benzaldehyde, and guaiacol | Aladdin (Shanghai, China) |

Table S1 Reagent Information

Note: The purity levels of the standard substances range from 80% to 99.5%.

Table S2 Qualitative analysis of aroma compounds

| No. | Compounds | CAS | RI^a^ | RI^b^ | RI^c^ | RI^d^ | Identification^e^ |
| --- | --- | --- | --- | --- | --- | --- | --- |
| 1 | Pyridine | 110-86-1 |  |  | 730 | 736 | MS, Std, RI |
| 2 | (+)-Limonene | 5989-27-5 | 1031 | 1035 |  |  | MS, Std, RI |
| 3 | 2-Picoline | 109-06-8 |  |  | 819 | 821 | MS, Std, RI |
| 4 | 6-Methyl-2-heptanone | 928-68-7 | 1216 | 1236 |  |  | MS, Std, RI |
| 5 | 2,6-Lutidine | 108-48-5 | 1248 | 1267 |  |  | MS, Std, RI |
| 6 | 3-Picoline | 108-99-6 |  |  | 847 | 861 | MS, Std |
| 7 | 6-Methyl-5-hepten-2-one | 110-93-0 | 1333 | 1340 |  |  | MS, Std, RI |
| 8 | Benzaldehyde | 100-52-7 | 1489 | 1502 |  |  | MS, Std, RI |
| 9 | 2-Methyl-1*H*-pyrrole | 636-41-9 |  |  | 841 | 856 | MS, Std |
| 10 | 5-Methylfurfural | 620-02-0 | 1594 | 1601 |  |  | MS, Std, RI |
| 11 | *β*-Cyclocitral | 432-25-7 | 1587 | 1598 |  |  | MS, Std, RI |
| 12 | Furfuryl alcohol | 98-00-0 | 1658 | 1666 |  |  | MS, Std, RI |
| 13 | 2(5*H*)-Furanone | 497-23-4 | 1752 | 1767 |  |  | MS, Std, RI |
| 14 | 4'-Methylacetophenone | 122-00-9 | 1780 | 1791 |  |  | MS, Std, RI |
| 15 | Ethyl phenylacetate | 101-97-3 | 1765 | 1779 |  |  | MS, Std, RI |
| 16 | 3-Acetylpyridine | 350-03-8 |  |  | 1109 | 1117 | MS, Std |
| 17 | Guaiacol | 90-05-1 | 1854 | 1862 |  |  | MS, Std, RI |
| 18 | Phenethyl alcohol | 60-12-8 | 1907 | 1922 |  |  | MS, Std, RI |
| 19 | Neophytadiene | 504-96-1 |  |  | 1828 | 1836 | MS, Std |
| 20 | *β*-Ionone | 14901-07-6 |  |  | 1483 | 1486 | MS, Std |
| 21 | 3,7,11,15-Tetramethyl-2-hexadecen-1-ol | 102608-53-7 |  |  |  |  | MS, Std |
| 22 | (-)-Ambroxide | 100679-85-4 |  |  |  |  | MS, Std |
| 23 | 2-Methoxy-4-vinylphenol | 7786-61-0 | 2143 | 2156 |  |  | MS, Std, RI |
| 24 | 3-Phenylpyridine | 1008-88-4 |  |  | 1461 | 1470 | MS, Std |
| 25 | Isophytol | 505-32-8 | 2316 | 2327 |  |  | MS, Std, RI |
| 26 | Dihydroactinidiolide | 17092-92-1 | 2341 | 2348 |  |  | MS, Std, RI |
| 27 | Indole | 120-72-9 | 2437 | 2441 |  |  | MS, Std, RI |
| 28 | Nicotine | 54-11-5 | 1859 | 1863 |  |  | MS, RI |
| 29 | Mesityl oxide | 141-79-7 |  |  | 789 | 797 | MS, Std, RI |
| 30 | *γ*-Butyrolactone | 96-48-0 |  |  | 905 | 908 | MS, Std, RI |
| 31 | Acetophenone | 98-86-2 |  |  | 1072 | 1078 | MS, Std, RI |
| 32 | 6-Methyl-3,5-heptadien-2-one | 1604-28-0 |  |  | 1084 | 1088 | MS, Std, RI |
| 33 | Isophorone | 78-59-1 |  |  | 1118 | 1122 | MS, Std, RI |
| 34 | 2,6,6-Trimethyl-2-cyclohexene-1,4-dione | 1125-21-9 |  |  | 1132 | 1139 | MS, Std, RI |
| 35 | (*E*)-5-isopropyl-8-methylnona-6,8-dien-2-one | 54868-48-3 |  |  | 1297 |  | MS, RI |
| 36 | *β*-Damascone | 35044-68-9 |  |  | 1406 | 1415 | MS, Std, RI |
| 37 | Geranylacetone | 3796-70-1 |  |  | 1451 | 1452 | MS, Std, RI |
| 38 | 4,7,9-Megastigmatrien-3-one II | 38818-55-2 |  |  | 1581 |  | MS, RI |
| 39 | 3-Hydroxy-*β*-damascone | 102488-09-5 |  |  | 1612 |  | MS, RI |
| 40 | 4,7,9-Megastigmatrien-3-one IV | 38818-55-2 |  |  | 1654 |  | MS, RI |
| 41 | *α*-Cyperone | 473-08-5 |  |  | 1768 | 1771 | MS, Std, RI |
| 42 | 2-Pentadecanone, 6,10,14-trimethyl- | 502-69-2 |  |  | 1841 | 1847 | MS, Std, RI |
| 43 | Farnesylacetone | 762-29-8 |  |  | 1917 | 1921 | MS, Std, RI |
| 44 | Cembrene | 1898-13-1 |  |  | 1930 | 1939 | MS, RI |
| 45 | Thunbergol | 25269-17-4 |  |  | 1971 |  | MS, RI |
| 46 | Sclareolide | 564-20-5 |  |  | 2069 |  | MS, Std |
| 47 | Sclareol | 515-03-7 |  |  | 2081 |  | MS, Std |
| 48 | Isoamyl acid | 503-74-2 |  |  | 861 | 867 | MS, Std, RI |
| 49 | 3-Methylvaleric acid | 105-43-1 |  |  | 939 | 941 | MS, Std, RI |
| 50 | Lactic acid | 50-21-5 |  |  |  |  | MS, Std |
| 51 | Benzoic acid | 65-85-0 |  |  | 1159 | 1170 | MS, Std, RI |
| 52 | Octanoic acid | 124-07-2 |  |  | 1167 | 1182 | MS, Std, RI |
| 53 | Phenylacetic acid | 103-82-2 |  |  | 1254 | 1262 | MS, RI |
| 54 | Succinic acid | 110-15-6 |  |  |  |  | MS, Std |
| 55 | Vanillic acid | 121-34-6 |  |  | 1585 | 1592 | MS, Std, RI |
| 56 | Myristic acid | 544-63-8 |  |  | 1759 | 1761 | MS, Std, RI |

Notes: RI^a^: the linear retention indices calculated from a series of n-alkanes (C_7_-C_30_) with DB-WAX column; RI^b^: retention indices referred to the literature value with DB-WAX column (http://webbook.nist.gov/chemistry/); RI^c^: the linear retention indices calculated from a series of n-alkanes (C_7_-C_30_) with DB-5MS column; RI^d^: retention indices referred to the literature value with DB-5MS column; Identification^e^: MS referred to identification by comparison with the NIST17 mass spectrometry database, RI referred to identification by comparison RI calculated in this study with literature values, Std referred to determination by standards.


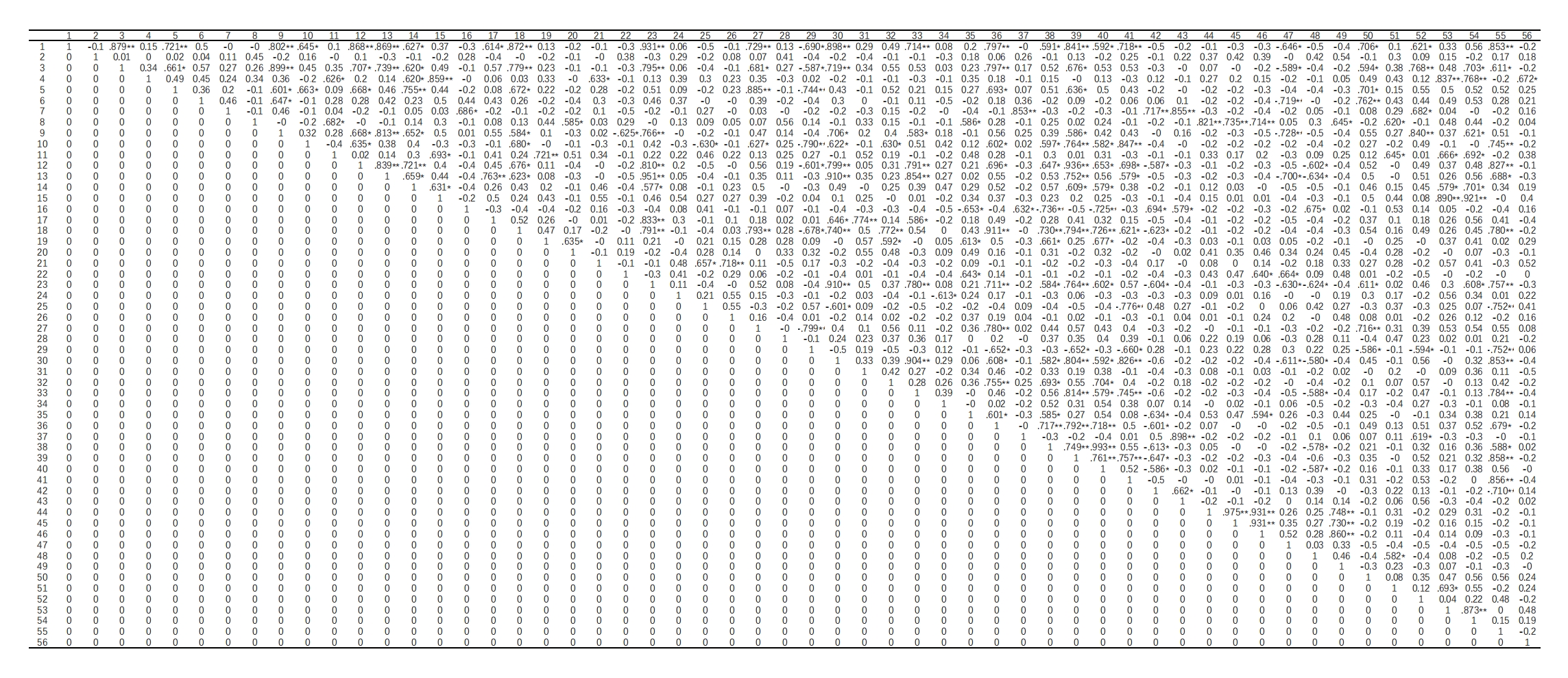
Table S3 Pearson correlation analysis of aroma compounds

Note: ‘*’ denotes differences were statistically significant at the 0.05 level; ‘**’ denotes differences were statistically significant at the 0.01 level.
